# Acute Esophageal Necrosis: A Successfully Managed Case

**DOI:** 10.7759/cureus.78499

**Published:** 2025-02-04

**Authors:** Francisca Carmo, João Miranda, Mariana Estrela, Raquel Moura, Jorge Reis, Pedro Magalhães

**Affiliations:** 1 Internal Medicine, Unidade Local de Saúde Gaia Espinho, Vila Nova de Gaia, PRT

**Keywords:** acute esophageal necrosis (aen), black esophagus, esophageal mucosa injury, high mortality, sepsis, upper gastrointestinal bleeding, upper gastrointestinal endoscopy (ogd)

## Abstract

Acute esophageal necrosis is a rare condition associated with a poor prognosis. It typically presents with upper gastrointestinal bleeding, and diagnosis is established via upper gastrointestinal endoscopy. Its etiology is often multifactorial and recommendations regarding its management and treatment are scarce and of low evidence level. We present the case of an 80-year-old male with multiple medical comorbidities, who presented to the Emergency Department with upper gastrointestinal bleeding associated with sepsis of an unknown origin. Upper gastrointestinal endoscopy revealed a necrotic and ulcerated esophagus in almost its entire extension, sparing only the proximal esophagus, which is consistent with stage I acute esophageal necrosis. He was managed with supportive care and discharged home in a stable condition. This report shows that early recognition and subsequent resuscitation are the keystones of management, regardless of diagnostic uncertainty.

## Introduction

Acute esophageal necrosis (AEN) is a rare condition with a poor prognosis. Its incidence is low (0.01-0.28%) [1,2]. It typically presents with upper gastrointestinal bleeding, and diagnosis is established via upper gastrointestinal endoscopy (UGE), which reveals segments of the esophageal mucosa exhibiting black discoloration, usually affecting the distal third and stopping abruptly at the gastroesophageal junction. Its etiology is often multifactorial, with a seemingly significant component of hypoperfusion and ischemia, coupled with alterations in the esophageal mucosal barrier. It is more prevalent in males, and its incidence increases with age, presenting a mortality rate of approximately 30% [3,1].

In the acute phase, perforation and recurrent gastrointestinal hemorrhage are the main complications, while in the subacute phase, the development of esophageal strictures can lead to dysphagia and the eventual need for intervention [1]. Despite some case reports, recommendations regarding its management and treatment are scarce and of low evidence level [4].

## Case presentation

An 80-year-old man, independent in activities of daily living, presented to the Emergency Department with hematemesis of two days' duration, associated with confusion and fever. He also reported a two-week history of food vomiting, dysphagia for liquids and solids, early satiety, and abdominal discomfort. He denied any other hemorrhagic losses such as melena, as well as other symptoms like retrosternal pain or dyspnea. The patient's relevant medical history included several vascular risk factors (hypertension controlled with triple therapy, dyslipidemia, and obesity), chronic kidney disease stage IIIa (baseline serum creatinine 1.4 mg/dL), atrial fibrillation, and benign prostatic hyperplasia. The patient and family denied any history of alcohol abuse. His medications included perindopril, indapamide, amlodipine, atenolol, rivaroxaban, fenofibrate, dutasteride, and tamsulosin.

On physical examination, the patient was obtunded, hypotensive (blood pressure 83/60 mmHg), tachycardic (heart rate 116 bpm), and febrile (38.2°C). His mucous membranes were colored, dehydrated, and showed white patches on the tongue and inner mouth cheeks; Cardiac and pulmonary auscultation was normal and the abdomen was soft and depressible, without tenderness on palpation. The remaining examination was unremarkable.

Laboratory results (Table 1) showed a significant elevation in C-reactive protein (24.2 mg/dL), acute kidney injury with a creatinine of 3.1 mg/dL and urea of 227 mg/dL, and hypokalemia (potassium 2.8 mmol/L). Muscle enzyme levels were slightly elevated (CK 219 U/L). Lactate levels and pancreatic enzymes were normal, and the patient did not present with anemia or coagulation abnormalities, in addition to the therapeutic levels of rivaroxaban.

**Table 1:** Laboratory studies results at the emergency department on admission

Laboratory Parameter	Result	Reference range
Hemoglobin (g/dl)	13.2	13.0-18.0
Leukocyte count (x10^3^/uL)	7.75	3.8-10.6
Platelet count (x10^3^/uL)	183	150-440
Creatinine (mg/dl)	3.13	0.67-1.17
Urea (mg/dl)	227	17-50
Sodium (mmol/L)	143	136-145
Potassium (mmol/L)	2.8	3.5-5.0
Total bilirubin (mg/dl)	1.48	0.1 - 1.1
Direct bilirubin (mg/dl)	0.96	0.1 - 0.3
Alanine transaminase (U/L)	63	4 - 50
Albumin (g/dl)	3.7	3.4 - 4.8
Total creatine kinase (U/L)	219	26 - 174
Pancreatic amylase (U/L)	53	13 - 53
Lipase (U/L)	23	13 - 60
C-reactive protein (mg/dl)	24.27	0-0.5
Lactate (mmol/L)	1.6	<2
Activated partial thromboplastin time (seg)	26.9	25.2 - 35.2
Prothrombin time (seg)	17.4	11.5 - 14.5
Rivaroxaban (ng/ml)	101.00	12-137

**Figure 1:**
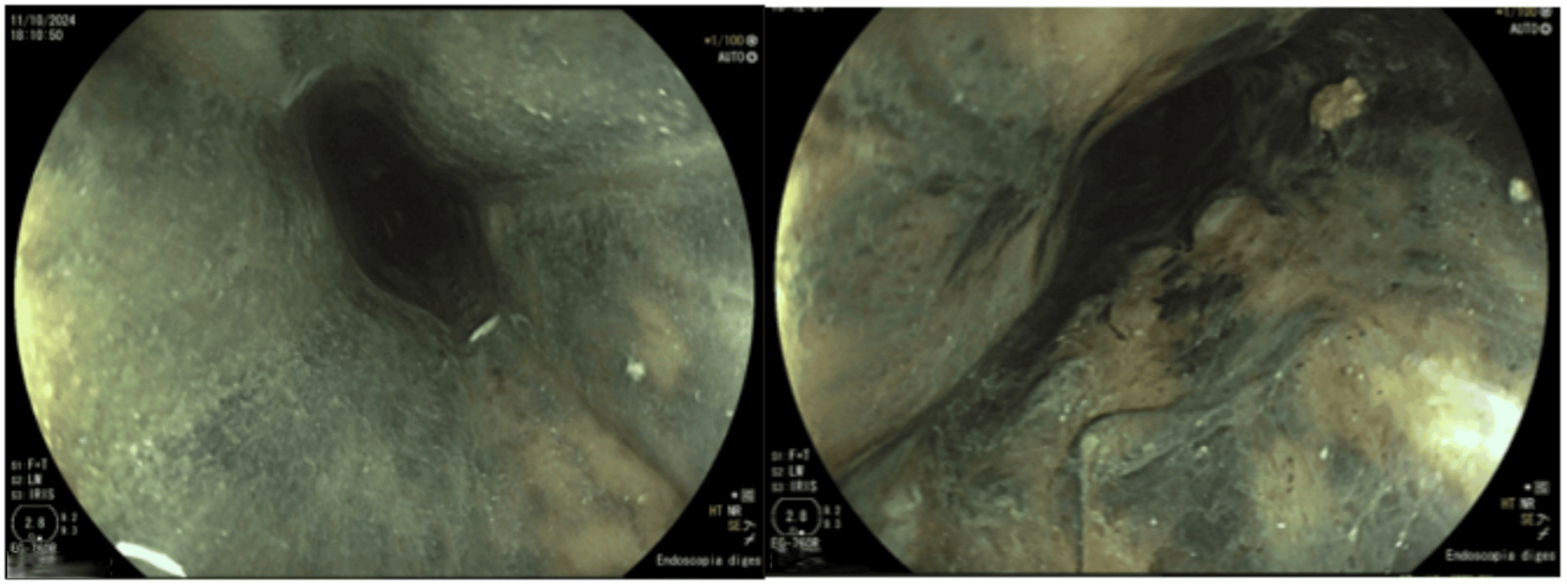
Upper gastrointestinal endoscopy at admission

Blood samples were collected for microbiology, and an upper gastrointestinal endoscopy (UGE) was performed, revealing a necrotic and ulcerated esophagus in almost its entire extension, sparing only the proximal esophagus, consistent with stage I AEN (Figure 1). Biopsies were not performed due to the risk of perforation.

**Figure 2:**
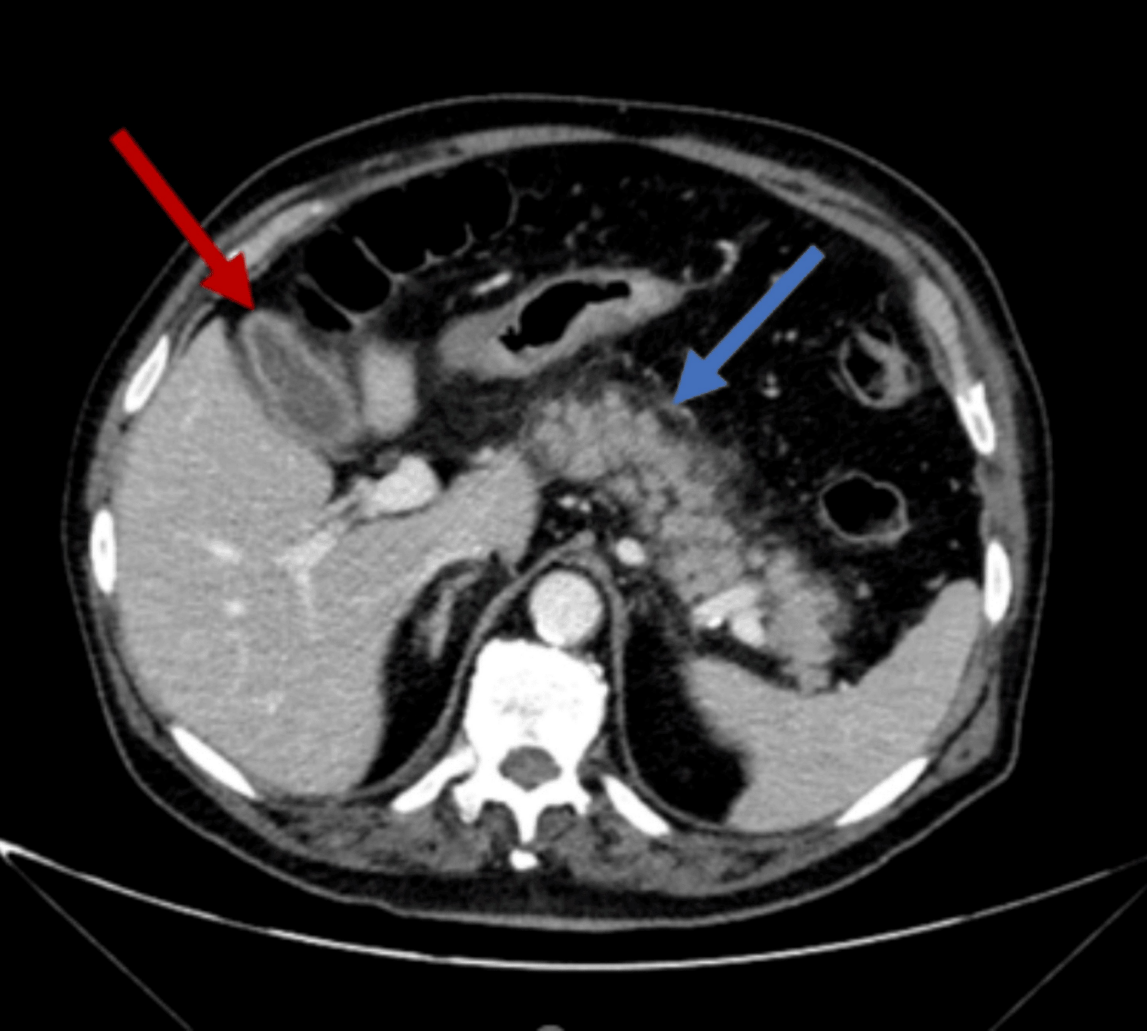
Abdominal CT scan (axial) reveals a slightly thickened gallbladder wall with pericholecystic fat stranding (red arrow), suggestive of cholecystitis. Additionally, the pancreas appears enlarged with peripancreatic fat stranding (blue arrow). No fluid collections or free fluid are seen within the peripancreatic space.

A computed tomography (CT) scan of the thorax, abdomen, and pelvis was also performed (Figure 2), suggesting the presence of cholecystitis/pancreatitis, which was not corroborated by laboratory studies or evaluation by General Surgery. Significant diffuse parietal thickening of the esophagus and densification of the surrounding fat were also visible, suggesting esophagitis.

The patient was admitted with AEN associated with sepsis of unknown source. Fluid resuscitation, electrolyte imbalance corrections, and broad-spectrum antibiotics with piperacillin-tazobactam and fluconazole were initiated. In addition, gastric acid suppression with intravenous pantoprazole 40 mg twice daily was initiated after an 80 mg bolus, and oral intake was withheld with total parenteral nutrition (TPN) started via a peripheral catheter.

The patient showed favorable clinical evolution, with sustained improvement in renal function and a significant reduction in inflammatory parameters, starting a clear liquid diet on the third day, with slow but progressive dietary advancement. Diet was well tolerated and no recurrence of upper gastrointestinal bleeding or other complications were noted. Adequate oral nutritional intake was achieved by day five at which point parenteral nutrition was suspended.

On the fifth day of hospitalization, he had a recurrence of fever, attributed to phlebitis associated with the peripheral venous catheter, with resolution after removal and local application of ice. Therefore, the patient completed seven days of piperacillin-tazobactam and 14 days of fluconazole.

Anticoagulation was started on the fifth day, with a low dose, subcutaneous enoxaparin 40 mg per day, and oral anticoagulation was restarted after 48 hours, without new bleeding.

During hospitalization, other risk factors, namely diabetes and occult malignancy, as well as other infections (human immunodeficiency virus, hepatitis B and C, and cytomegalovirus) were excluded.

**Figure 3:**
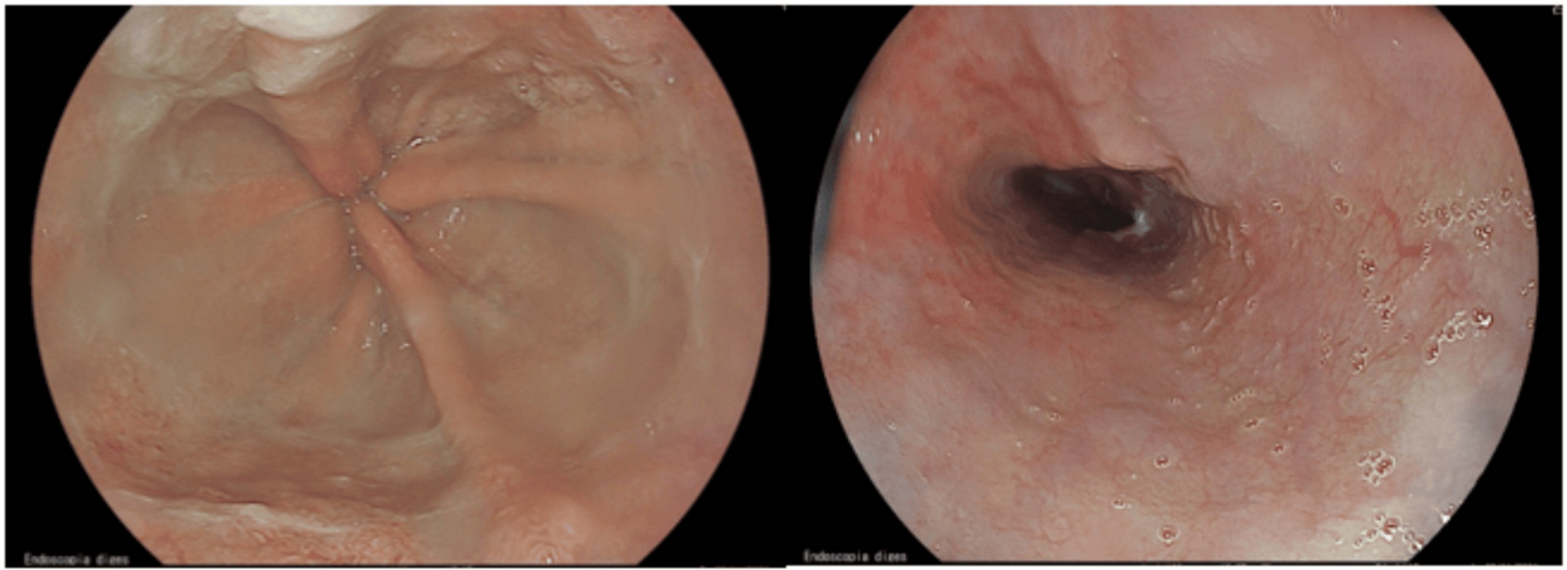
Upper gastrointestinal endoscopy after one month

The patient was discharged on the 10th day of hospitalization, with a re-established diet, and maintained acid suppression with pantoprazole 40 mg twice daily and with adjustment of his home medications, particularly with suspension of antihypertensive therapy given his controlled blood pressure during hospitalization. After one month, a repeat UGE was performed, showing the resolution of all findings and no evidence of strictures (Figure 3). At three months, there was no recurrence of the episode or complications.

## Discussion

AEN is a rare condition associated with high mortality, therefore requiring prompt multidisciplinary intervention. Upper endoscopy reveals diffuse circumferential black discoloration of the esophageal mucosa. While the distal third is most commonly affected, necrosis can extend proximally, as observed in this case.

The etiology of AEN is likely multifactorial and risk factors included include male sex (four more times likely to occur in males), advanced age, alcohol abuse, diabetes mellitus, hypertension, cardiovascular disease, prothrombotic conditions, chronic kidney disease, malignancies, and gastroesophageal reflux disease [1-7]. In the present case, no specific cause for the AEN was found. It was likely a combination of esophageal mucosa injury from vomiting, coupled with tissue hypoperfusion from sepsis in a susceptible patient (male sex, advanced age, arterial hypertension on multiple drugs, and chronic kidney disease). Other factors such as occult tumor or gastric obstruction were excluded by CT scan and upper endoscopy.

Although biopsy is recommended for confirmation and to exclude other conditions, it is not necessary to establish the diagnosis [1,5]. In the current case, while a biopsy could have aided in the etiological diagnosis and treatment planning, it was deemed too risky due to potential bleeding and perforation, therefore it was not performed. Despite the absence of a clear source of infection and negative blood cultures, given the severe presentation and the existence of oral candidiasis, antibiotic therapy with piperacillin-tazobactam and fluconazole was maintained.

Management of AEN is primarily medical but can include surgical intervention in the event of complications such as perforation and mediastinitis. Initial treatment often involves resuscitation with fluids and blood components and intravenous proton pump inhibitors to suppress gastric acid production and reduce local insult to the vulnerable esophagus [1-7]. Sucralfate use has also been described. Implementation of nothing-by-mouth order with TPN appears to be associated with improved outcomes [1,5-7]. The perfect timing to transition to a soft diet is unknown. Some reviews recommend withholding oral intake for 24 hours, whereas others suggest at least one week [3,4]. Dietitians are crucial in managing good nutritional support. In this case, the initiation of TPN within the first days was crucial for the patient's favorable recovery, preventing malnutrition and supporting their high metabolic needs during this critical period. Due to the patient's rapid improvement, we started a clear liquid diet on day three, advancing it gradually. This approach was successful, and we were able to stop parenteral nutrition by day five.

Follow-up endoscopy is recommended after approximately one month to confirm mucosal healing and rule out complications such as strictures and fistulae [3,5-7]. In this specific case, given the lack of a biopsy during the initial diagnosis, endoscopy was crucial to exclude underlying lesions. 

## Conclusions

AEN is an uncommon condition that can present in several different ways, including upper gastrointestinal bleeding, signs of sepsis, or peripheral hypoperfusion. Early recognition and subsequent resuscitation are the keystones of management, regardless of diagnostic uncertainty. This case report highlights the challenges in diagnosing and managing this condition, emphasizing the importance of addressing the multiple risk factors and underlying comorbidities.

A multimodal treatment strategy, prioritizing aggressive mucosal protection through proton pump inhibitors, a strict nothing-by-mouth regimen with total parenteral nutrition, alongside comprehensive supportive care, may significantly improve clinical outcomes in cases of AEN.
